# Riverine Realities: Evaluating Climate Change Impacts on Habitat Dynamics of the Critically Endangered Gharial (*Gavialis gangeticus*) in the Indian Landscape

**DOI:** 10.3390/ani15060896

**Published:** 2025-03-20

**Authors:** Imon Abedin, Hilloljyoti Singha, Shailendra Singh, Tanoy Mukherjee, Hyun-Woo Kim, Shantanu Kundu

**Affiliations:** 1 Department of Zoology, Bodoland University, Kokrajhar 783370, India; 2 Centre for Wildlife Research and Biodiversity Conservation, Bodoland University, Kokrajhar 783370, India; 3 Turtle Survival Alliance Foundation India (TSAFI), Lucknow 226021, India; 4 Agricultural and Ecological Research Unit, Indian Statistical Institute, Kolkata 700108, India; 5 Department of Marine Biology, Pukyong National University, Busan 48513, Republic of Korea; 6 Marine Integrated Biomedical Technology Center, National Key Research Institutes in Universities, Pukyong National University, Busan 48513, Republic of Korea; 7 Department of Biology, Faculty of Science and Technology, Airlangga University, Surabaya 60115, Indonesia; 8 Ocean and Fisheries Development International Cooperation Institute, College of Fisheries Science, Pukyong National University, Busan 48513, Republic of Korea; 9 International Graduate Program of Fisheries Science, Pukyong National University, Busan 48513, Republic of Korea

**Keywords:** freshwater ecosystem, crocodilia, threatened species, habitat dynamics, conservation, South Asia

## Abstract

The endemic and critically endangered gharial, *Gavialis gangeticus,* experienced a severe population decline in its range. However, conservation efforts, notably through the implementation of “Project Crocodile” in India, have led to a significant recovery of its population. The present study employs an ensemble Species Distribution Model (SDM) to delineate suitable habitats for *G. gangeticus* under current and future climatic scenarios to understand the impact of climate change. The model estimates that 46.85% of the area of occupancy is suitable under the present scenario, with this suitable area projected to increase by 145.16% in future climatic conditions. States such as Madhya Pradesh, Uttar Pradesh, and Assam are projected to experience an increase in habitat suitability, whereas Odisha and Rajasthan are anticipated to face declines. The study recommends conducting ground-truthing ecological assessments using advanced technologies and genetic analyses to validate the viability of newly identified habitats in the Lower Ganges, Mahanadi, and Brahmaputra River systems. These areas should be prioritized within the Protected Area network for potential translocation sites allocation. Collaborative efforts between the IUCN-SSC Crocodile Specialist Group and stakeholders are vital for prioritizing conservation and implementing site-specific interventions to protect the highly threatened gharial population in the wild.

## Simple Summary

The critically endangered and endemic gharial (*Gavialis gangeticus*), once on the edge of extinction, has been revived in the wild through focused conservation efforts across the Indian subcontinent. Today, gharial populations are restricted to fragmented habitats in northern India and lowland Nepal. Despite extensive research on the species, a significant knowledge gap persists regarding the effects of climate change on its spatial distribution. This study aims to address this gap by analyzing the impacts of climate change on the gharial’s habitat suitability and long-term viability. The findings will provide crucial insights into habitat dynamics and ecological requirements, contributing to the species’ long-term conservation and protection in the wild.

## 1. Introduction

The Earth is approaching a critical threshold of irreversible biodiversity loss, driven by the ongoing human-induced mass extinction event [1]. This accelerated species decline is primarily attributed to anthropogenic factors such as habitat destruction, overexploitation, and climate change [2]. Consequently, the biodiversity of freshwater ecosystems, which cover only 1% of the Earth’s surface yet support one-third of all vertebrate species, have lost over 85% of their habitats [3,4,5]. Notably, the freshwater reptiles inhabiting these ecosystems, including crocodilians, turtles, and snakes, are integral components of aquatic environments, contributing significantly to both biodiversity and ecological stability [6,7]. These species fulfill diverse ecological roles, functioning as apex predators, scavengers, etc., thereby influencing trophic dynamics and facilitating nutrient cycling [8,9]. However, the freshwater ecosystem faces serious threats like water pollution, hydrological alterations, habitat degradation, and proliferation of invasive species [10]. The continued and intensifying anthropogenic pressure on this ecosystem poses a significant risk for world biodiversity, especially to the megafauna species [10,11]. In particular, these freshwater larger-bodied taxa are at a disproportionately elevated risk of population declines and potential extinctions, with a trend that is projected to persist or intensify in the coming years [12,13]. The freshwater resources in tropical regions are highly susceptible to the effects of climate change with regional variations [14]. Particularly, river systems originating from the Himalayan region, such as the Indus, Ganges, and Brahmaputra, face additional environmental and anthropogenic pressures such as flash flood and frequent course alteration due to rapid glacial melt and erratic monsoons, high population demand, extreme sedimentation, etc. [15]. These river basins play a critical role in maintaining biodiversity and supporting a diverse array of wildlife populations in the Indian subcontinent [16,17].

In this ecosystem of the Indian Subcontinent, the gharial (*Gavialis gangeticus*), an endemic and critically endangered species under the family Gavialidae, inhabits the region [18,19]. It is an ectothermic species and is considered one of the rarest aquatic animals, serving as both a flagship and keystone species in its freshwater ecosystem [20,21,22]. It is easily distinguishable from other crocodilian species due to its unique morphological traits, including an elongated, narrow snout and a prominent, hollow, bulbous nasal protuberance known as the “Ghara” [23]. The gharial has the most restricted distribution range and is listed as the most threatened species of all crocodilians in the Indian subcontinent [24,25]. This species is a river-dwelling crocodilian known for its extensive use of river systems, exhibiting seasonal migratory behavior and complex social hierarchies. The female nest on seasonally exposed sandbanks along slow-moving stretches of medium to large rivers, typically laying an average of 40 eggs. As monsoon waters rise, adult gharials guarding the nests embark on long-distance seasonal migrations. During this time, crèches disband, and hatchlings disperse widely into aquatic shoreline habitats [26].

Historically, the gharial was reported across the major river systems in the Indian subcontinent, including the Indus, Ganges, Mahanadi, Brahmaputra-Meghna, and possibly the Irrawaddy, with an extent of 80,000 sq. km [24]. The estimated population ranged between 5000 and 10,000 individuals until the 1940s [27]. However, the population of this species declined by more than 80% due to habitat loss, poaching, and accidental deaths from passive fishing in the past century [18,28]. Additionally, major water control structures, such as dams and barrages, along with irrigation projects and sewage discharge, have significantly contributed to the population decline of this species, as assessed by the IUCN-SSC Crocodile Specialist Group (CSG) [29]. As a result, the species is now extinct in countries such as Myanmar, Bhutan, and Pakistan [30,31]. During the early 1970s, the gharial was confined to a few isolated populations in the Ganges and its main tributaries, Mahanadi and Brahmaputra, in the Indian subcontinent [32]. As a result, the gharial is now classified as ‘Critically Endangered’ on the IUCN Red List and listed in ‘Appendix I’ of Convention on International Trade in Endangered Species of Wild Fauna and Flora (CITES) [29,33].

In recognition of their vulnerability, the Government of India placed all three species of crocodilians viz. the Indian mugger crocodile (*Crocodylus palustris*), the saltwater crocodile (*Crocodylus porosus*), and the gharial under ‘Schedule I’ of the Indian Wildlife (Protection) Act, 1972 [18,34]. Thus, to support the conservation of crocodiles in India, a captive breeding program (Project Crocodile) was initiated in 1975 for all three species found in the country [34]. This program was designed to protect these species and facilitate the recovery of their depleted populations. The conservation strategies included ’head-starting’, assisted translocation programs, and the protection of suitable natural habitats to support the gharial population’s restoration in the wild [23]. These efforts have been instrumental in preventing the immediate extinction of the gharial and have contributed to its recovery [25,35]. Despite the massive decline, the gharial population has begun to rebound due to these targeted conservation initiatives initiated in the mid-1970s [30]. Subsequently, numerous studies have been conducted to examine the behavioral patterns, occupancy, genetics, and ecology of the gharial in the Indian subcontinent [2,24,29,35,36,37,38,39,40]. However, amidst the ongoing climate crisis, there is a notable gap in research regarding the impact of climate change on this reptilian species [41,42]. This critical knowledge gap must be addressed, as climate change may disrupt physiological and ecological processes, degrade habitat quality, alter prey dynamics, heighten disease susceptibility, and intensify human–wildlife conflicts. This will assist in planning and supporting ongoing conservation efforts in the context of climate change, while also establishing a designated management plan for the future.

In this context, species distribution models (SDMs) have emerged as valuable tools for enabling precise predictions of current habitat conditions and future climatic projections for species [43,44,45,46]. These models utilize existing data on species and their ecological niches across both spatial and temporal scales to provide a deeper understanding of habitat dynamics in respect to climate and habitats [47,48]. This ensemble method utilizes a combination of modeling algorithms to forecast species distributions within different geographic regions and timelines, leveraging the distinct advantages of each model to address various factors that influence distribution patterns [49]. By integrating the strengths and mitigating the limitations of individual models, this approach improves the precision and reliability of the distribution predictions. Therefore, this study employs ensemble SDMs to: (a) identify the current extent of suitable habitats within the Ganges-Brahmaputra-Mahanadi River basins in India, (b) project suitability dynamics under future climatic scenarios and possible centroid shift from its present extent, (c) assess habitat quality and shape geometry in both current and future contexts. The findings from this study will be instrumental in guiding and enhancing ongoing conservation efforts, particularly in prioritizing conservation areas within the gharial’s largest habitat range. In addition, the area delineated by the study during the future climate change scenario will be instrumental in directing the prioritized areas for conservation and further studies.

## 2. Materials and Methods

### 2.1. Study Area and Species Occurrence Records

**Figure 1:**
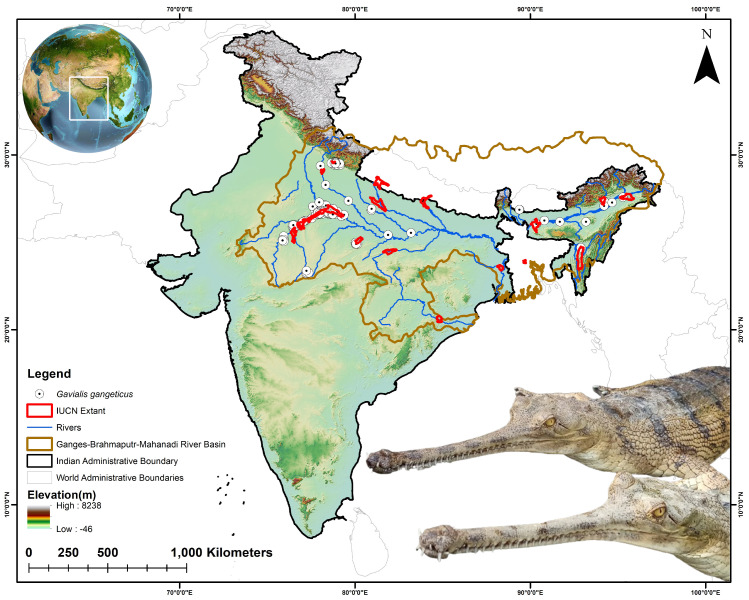
The map illustrates the entire IUCN designated range of the gharial, along with presence locations obtained from primary field surveys and secondary sources. The map also highlights the training area of the model, i.e., the Ganges, Brahmaputra, and Mahanadi River basins. The photograph of the gharial was captured by the third author (S.S.).

Currently, gharials are confined to only 14 isolated and restricted locations in the Gangetic plains of northern India and lowland Nepal [33]. As of 2018, the species has been extirpated from Pakistan, Bhutan, Myanmar, and potentially Bangladesh, where only a few stray individuals may survive [29]. The largest gharial populations are now concentrated in the Ganges-Brahmaputra and Mahanadi River basins in India, which were therefore selected as the training area for model development (Figure 1). For the collection of presence locations, the ecologist team from the Turtle Survival Alliance Foundation India (TSAFI) conducted primary field surveys in the Upper Ganges (*n* = 1), Ghaghara (*n* = 9), and Chambal (*n* = 26) rivers (Table S1). These surveys resulted in a total of 36 opportunistic sightings, comprising both adult individuals and nesting sites. In addition, the study also utilized 785 occurrence points obtained from the GeoCAT website, which aggregates data from secondary resources such as GBIF and iNaturalist, accessed on 02 November 2024 [50]. To ensure dataset reliability and ecological relevance, records of museum specimens and captive individuals were not taken into consideration. Correspondingly, 32 location points were derived from previous studies [38,51,52,53]. The spatial correlation of occurrence data was analyzed at a resolution of 1 sq. km using SDM toolbox v2.4 [54]. This resolution was taken to match the pixel size of the raster data, minimizing overfitting and enhancing model accuracy. The final model was based on the aggregate of 403 presence points for the gharial within the defined training extent.

### 2.2. Selection of Covariates

To identify suitable habitat patches for gharials in the study area, a combination of bioclimatic, habitat, and anthropogenic variables was utilized [55]. The standard set of 19 bioclimatic variables was obtained from the WorldClim database (https://www.worldclim.org/, accessed on 2 November 2024) and extracted for use within the study area (Figure S1) [56]. Given that gharials primarily inhabit freshwater ecosystems and riparian zones, the Euclidean distance to waterbodies was included as a key variable [33]. This was calculated using the Euclidean distance function in ArcGIS v. 10.8 by isolating the waterbody class from the Land Use Land Cover raster of the ESRI Sentinel-2 10-Metre dataset, sourced from the ESRI Living Atlas website (https://livingatlas.arcgis.com/landcover/, accessed on 2 November 2024) [57]. The dataset tiles were merged to encompass mainland India to ensure comprehensive spatial coverage. In addition, the Global Human Footprint Dataset was used as the anthropogenic predictor to analyze the Human Influence Index (HII) and assess the degree of human impact on the species [58]. All spatial variables were standardized to a resolution of 30 arc-seconds (~1 sq. km) using the spatial analyst extension in ArcGIS v. 10.6. Thus, to ensure analytical robustness, spatial multicollinearity testing was performed using the SAHM (Software for Assisted Habitat Modeling) package within the VisTrails software [59]. The variables with a Pearson correlation coefficient (r) exceeding 0.8 were excluded from further analysis to minimize redundancy and enhance model reliability (Figure S1) [60]. These same variables were evaluated using Spearman and Kendall correlation coefficients to further reduce the likelihood of inter-variable correlation. Consequently, based on these three-correlation metrics, variables demonstrating higher importance and exhibiting a correlation coefficient below 0.8 were selected for the final modeling process based on standard protocol [61,62,63,64] (Figure S1).

Notably, to evaluate the potential impacts of climate change, the study analyzed future scenarios under two Shared Socioeconomic Pathways (SSPs): SSP245 and SSP585, for the periods 2041–2060 and 2061–2080. The future climate projections utilized the HadGEM3-GC31 LL model, part of the Coupled Model Intercomparison Project Phase 6 (CMIP6). This model was selected for its proven reliability in simulating climate variability and temperature trends across South and Southeast Asia [65,66]. In addition, to focus exclusively on the effects of climatic changes on the species’ distribution, non-climatic variables were held constant during the future climate analyses. This approach ensured that projections remained ecologically relevant to gharial habitats [67].

### 2.3. Ensemble Model Utilization

To develop the final distribution model for this species, multiple modeling algorithms were combined using an ensemble approach. Primarily, four distinct algorithms—Boosted Regression Tree (BRT), Generalized Linear Model (GLM), Multivariate Adaptive Regression Splines (MARS), Maximum Entropy (MaxEnt), and Random Forest (RF)—were applied [47,68,69]. These algorithms were run using the SAHM package within the VisTrails software [59,70]. The output consisted of probability surfaces ranging from “0” (lowest suitability) to “1” (highest suitability), with binary maps generated by applying the minimum training presence threshold. An ensemble count map was created on a scale from “0” to “5”, where each pixel represented the number of models in agreement. A value of “5” indicated consensus across all four models, providing a basis for analyzing habitat configuration. The pixels classified as “5”, where all models unanimously agreed, will be considered as the suitable habitat/area for the species. This area will be selected for analysis to ensure a comprehensive evaluation of its ecological significance. In addition, the model performance was evaluated and compared using multiple metrics, including AUC, True Skill Statistic (TSS), Cohen’s Kappa, Proportion Correctly Classified (PCC), specificity, and sensitivity. These metrics were calculated for both the training datasets and cross-validation sets (*n* = 10) [71,72,73,74]. Likewise, the centroid shift for gharial, from the current scenario to future SSP projections across both timeframes, was evaluated utilizing the centroid change function within SDM Toolbox v2.4.

### 2.4. Assessment of Habitat Quality and Shape Geometry

The qualitative and geometric characteristics of suitable gharial habitat patches within the Ganges, Brahmaputra, and Mahanadi River Basins were analyzed under both current and projected future scenarios to facilitate comparative analyses. This evaluation was conducted using class-level metrics in FRAGSTATS version 4.2.1, a tool widely utilized in landscape ecology and environmental management [75]. FRAGSTATS provides a comprehensive suite of metrics and indices to assess and interpret the spatial patterns, geometry, and composition of landscapes [76]. The analysis incorporated metrics such as the number of patches (NP), patch density (PD), total edge (TE), largest patch index (LPI), aggregate index (AI), and landscape shape index (LSI). Metrics like NP, PD, TE, and LPI provided insights into the size, density, and edge characteristics of habitat patches within the study area. The LSI assessed the complexity of patch shapes, indicating the degree of irregularity in their geometry. Meanwhile, the AI metric quantified the proximity and clustering of habitat patches, offering a measure of their aggregation or dispersion across the landscape. These metrics collectively enabled a detailed evaluation of the spatial dynamics and configuration of gharial habitats [45].

## 3. Results

### 3.1. Model Validation and Variable Impact

**Figure 2:**
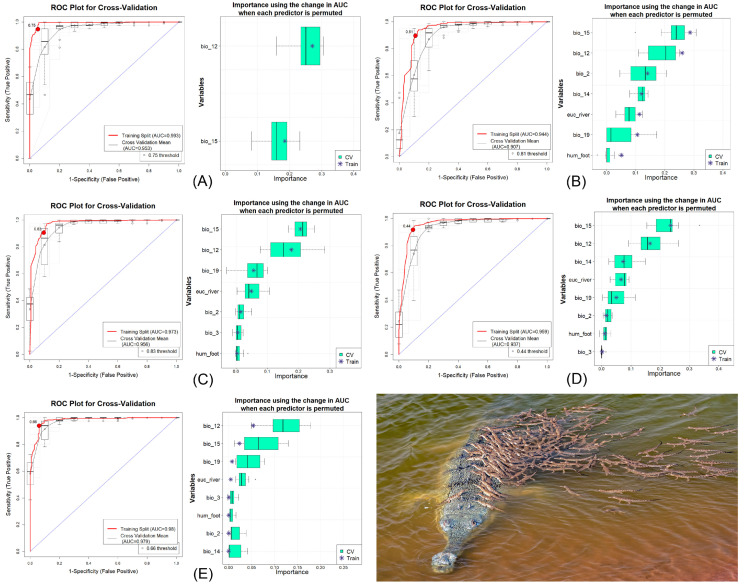
Model evaluation plots depicting the average training ROC for both training and cross-validation (CV), along with the predictors selected by the model across replicate runs under five different models. (**A**) Boosted Regression Tree (BRT), (**B**) Generalized Linear Model (GLM), (**C**) Multivariate Adaptive Regression Splines (MARS), (**D**) Maximum Entropy (MaxEnt), and (**E**) Random Forest (RF). The photograph of the gharials was captured by Dhritiman Mukherjee.

The ensemble model demonstrated notable performance for both the training and cross-validation datasets, yielding consistently high values (Figure 2, Figures S2 and S3). Among the individual algorithms, MaxEnt utilized all eight predictors, while BRT used only two out of the eight. The training AUC values ranged from 0.944 to 0.993, while cross-validation AUC values ranged from 0.907 to 0.979 (Table 1). The fact that AUC values exceeded 0.75 for both training and cross-validation sets further confirmed the strong performance of the ensemble model. The GLM exhibited the largest ΔAUC value (0.037), whereas RF recorded the smallest ΔAUC value (0.001). The high performance of the model run was validated by high values for other metrics, such as the True Skill Statistic (TSS), Proportion Correctly Classified (PCC), Cohen’s Kappa, sensitivity, and specificity. These metrics collectively underscored the robustness and reliability of the ensemble model. The most influential variable determining gharial habitat suitability in the study area was identified as annual precipitation (bio_12), with a percentage contribution of 33.75%, closely followed by precipitation seasonality (bio_15) at 33.70% (Figure 2, Table 2). Notably, the Euclidean distance to waterbodies (euc_water) contributed 8.53% to habitat suitability, whereas the anthropogenic variable, Human Influence Index (hum_foot), accounted for 2.4% of the model’s contribution.

### 3.2. Spatiotemporal Habitat Suitability Dynamics and Centroid Shift

**Figure 3:**
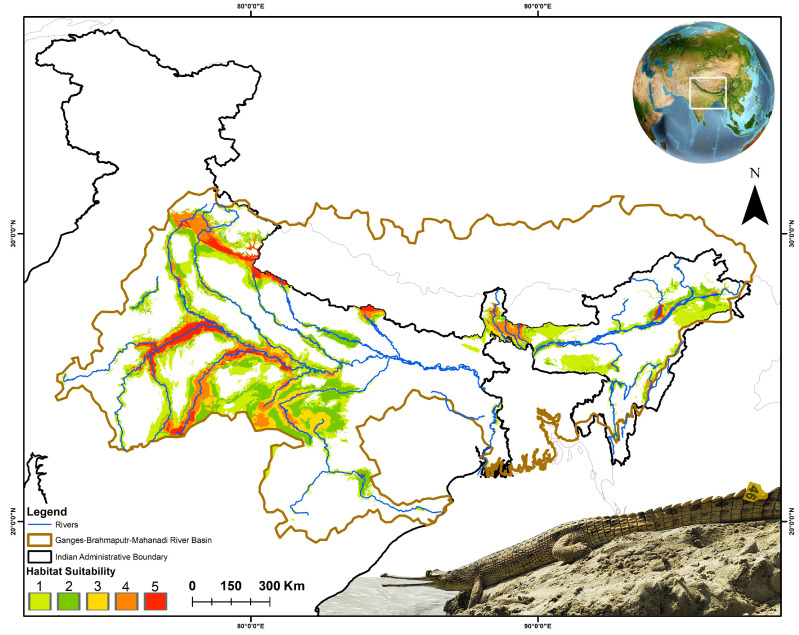
The map illustrates the suitable areas within the training region under the present scenario. The count map represents the model agreement, with a count of ‘5’ indicating the highest level of agreement among all five models, highlighting the most suitable areas. The photograph of the gharials was captured by third author (S.S.).

The distribution model identified a total of 37,487 sq. km as suitable habitat for the gharial within the Ganges-Brahmaputra-Mahanadi River basins in India under the current scenario (Figure 3, Table S2). Among the range states within these basins, Uttar Pradesh stands out as having the largest suitable area, encompassing approximately 10,954 sq. km, followed closely by Madhya Pradesh (10,797 sq. km) (Table 3). Consequently, other Indian states, including Rajasthan, Uttarakhand, Assam, and Bihar, have also been identified as having significant areas of suitable habitat for this species. Interestingly, the projections indicate that this suitable habitat extent is expected to expand across the study area in both timeframes under the two future climatic scenarios. In total, 20 Indian states have been identified as harboring a suitable habitat for this species, either under current conditions or in future scenarios.

**Figure 4:**
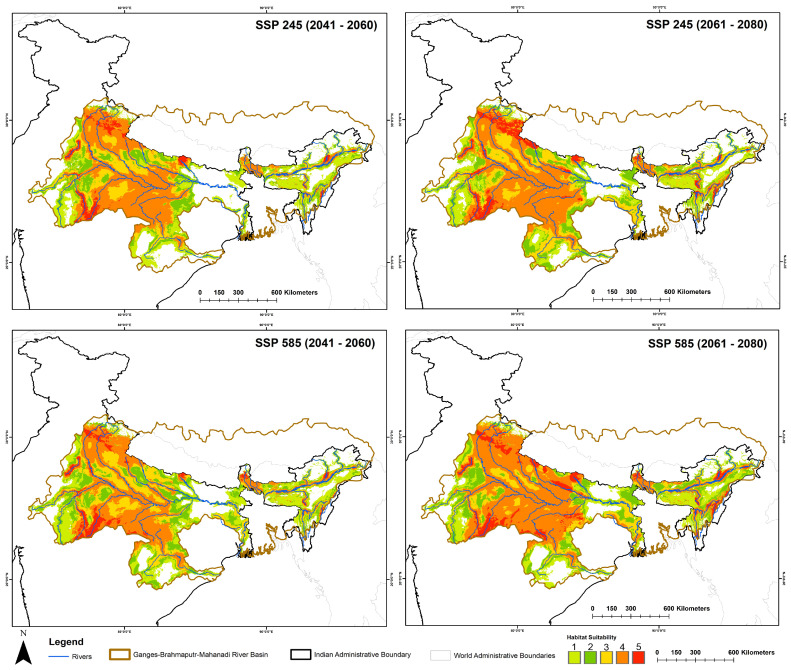
The map illustrates the suitable areas within the training region under different future climatic scenarios. The count map represents the model agreement, with a count of ’5’ indicating the highest level of agreement among all five models, highlighting the most suitable areas.

The projected suitable habitat range for this species is expected to expand by 36.42% to 145.16% under future climatic scenarios compared to the current extent (Figure 4, Table 3). Within the SSP245 scenario, the most pronounced increase is observed during the 2061–2080 timeframe. The state of Madhya Pradesh is projected to exhibit a 73.27% increase in suitable habitat during 2041–2060, rising to 131.80% by 2061–2080. Similarly, significant expansions are anticipated in Assam, Uttarakhand, and Bihar, with increases of up to 165.01%, 316.87%, and 188.51%, respectively. The state of Haryana demonstrates the highest gain, with its suitable habitat expanding from 70 sq. km at present to 2418 sq. km by 2061–2080. Interestingly, Uttar Pradesh is projected to experience a 47.85% reduction in suitable habitat during the 2041–2060 timeframe; however, this is followed by a recovery, with a 33.79% increase by 2061–2080 compared to the present scenario. Notably, Odisha is expected to lose its entire suitable habitat extent in both timeframes under the SSP245 scenario.

In the SSP585 climatic scenario, projections indicate a consistent trend of increasing habitat suitability for the species across both timeframes (Figure 4, Table 3). This scenario demonstrates the most significant expansion of suitable habitat within the study area compared to other scenarios. Madhya Pradesh is predicted to retain the largest suitable habitat area, with estimates of 32,241 sq. km in the 2041–2060 timeframe and 31,150 sq. km in the 2061–2080 timeframe. These values highlight the central role of Madhya Pradesh in the conservation of this species under changing climatic conditions. Specifically, other states that have not traditionally been associated with high suitability for the species, such as Arunachal Pradesh, Manipur, Jharkhand, and Chhattisgarh, are projected to exhibit notable increases in suitable habitat during the 2061–2080 timeframe in the SSP585 future climatic scenario.

**Figure 5:**
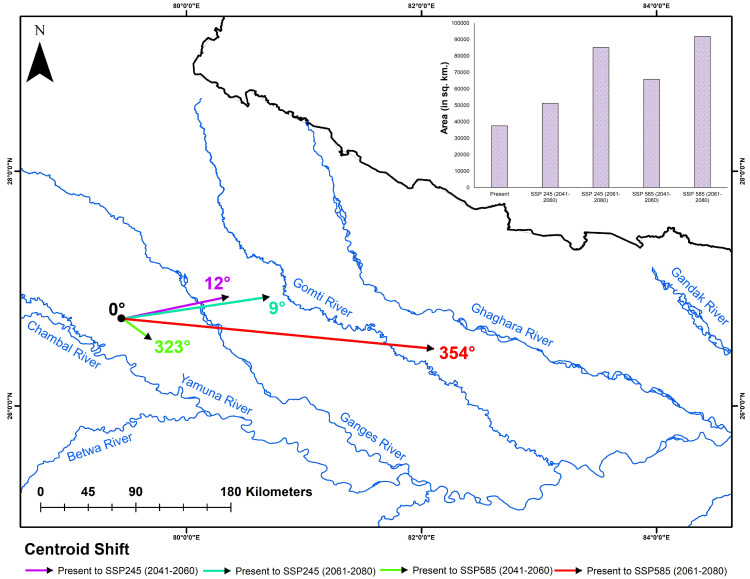
The map displays the major rivers and the centroid shift under future scenarios compared to the present. The inset bar chart represents the suitable area (in sq. km) under the present and various future climatic scenarios.

Furthermore, the analysis of centroid shifts under projected future climatic scenarios, compared to the present, indicated a notable movement of suitable habitat areas toward eastern India (Figure 5). This directional shift varied across scenarios and timeframes, ranging from 9° to 354° relative to the current centroid location. Specifically, the smallest observed shift, measured at 9°, occurred under the SSP245 scenario for the 2061–2080 timeframe. Conversely, the largest shift, measuring 354°, was observed under the SSP585 scenario for the same timeframe.

### 3.3. Spatial Configuration and Geometry

The analysis of habitat shape geometry provides valuable insights into priority areas under both SSP245 and SSP585 scenarios across the evaluated timeframes (Table 4). The results reveal a significant increase in the NP in future scenarios when compared to the present, with increments ranging from 44.32% to 96.13%. This increase in the NP suggests a notable alteration in the spatial configuration of suitable habitats. It is also linked to their denser configuration, as indicated by the rise in PD of up to 97.37%. The evaluation also demonstrated an increase in the size of the largest habitat patches, as indicated by the LPI, which exhibited an increase of up to 33.94%. This suggests that the largest suitable patches in the future scenarios occupy a greater proportion of the landscape compared to the present scenario. The increase in TE by 69.6% to 169.21% and in the LSI by 35.26% to 59.12% indicates that habitat patches in the future scenarios are characterized by greater edge lengths and more complex geometries. This increase in edge length and shape complexity suggests a shift in the structural properties of the habitat patches. Despite these changes in patch size, density, and shape, the AI that measures the proximity and clustering of habitat patches showed minimal variation across the scenarios. This stability in AI suggests that the spatial arrangement and connectivity of habitat patches remained relatively consistent between the current and future scenarios. These metrics collectively provided a comprehensive assessment of changes in habitat geometry under future SSP245 and SSP585 scenarios, offering a detailed characterization of shifts in spatial patterns and configurations of suitable gharial habitats.

## 4. Discussion

The conservation initiatives in recent decades have played a pivotal role in mitigating the risk of extinction for numerous threatened species [77,78]. While these efforts are widely recognized and commonly employed to achieve specific conservation objectives, there are cases where they fail to meet the desired goals of establishing or restoring populations [79]. The incorporation of adaptive conservation measures, combined with detailed knowledge of the key biological and ecological traits of target species, has proven essential for addressing conservation challenges [77]. Additionally, robust monitoring frameworks also play a crucial role in identifying shortcomings and devising effective strategies to enhance the success of such programs. In addition, the identification and prioritization of critical areas are fundamental to supporting targeted species in any conservation endeavor [80]. This is specifically important because certain prioritized areas may inadvertently be overlooked due to the absence of evidence indicating species presence, which does not necessarily imply the absence of the species [81]. Accordingly, the IUCN-SSC groups also highlight the significance of integrated ecological studies and varied perspectives in evaluating species for inclusion in the Red List of Threatened Species database. In such scenarios, SDMs are invaluable as they identify environmental conditions similar to known species habitats, even in areas where the species may not yet have been observed. These identified extents represent potential target areas that require ground-truthing to confirm their suitability before implementing conservation interventions. Hence, this study identified suitable habitats for the threatened gharial and prioritized areas for conservation under potential future climatic scenarios, providing a scientific basis for informed and effective conservation planning. This highlighted areas where the species may currently be absent but could be considered as potential translocation sites following on-the-ground ecological assessments. The estimated area identified by the suitability model under the present scenario accounts for 46.85% of the area of occupancy for the gharial [29]. However, it is important to note that the delineated area in this study does not necessarily confirm the species’ presence but rather indicates regions with similar ecological and environmental envelopes to the known niche of the gharial. This projected increase in suitable habitat under future climatic scenarios may suggest that the gharial could withstand, adapt to, or recover from the impacts of climate change in the future. This observed range expansion may also be influenced by climatic shifts, as the most significant predictors in the model were climatic variables—namely, Annual Precipitation (bio_12) with a 33.75% contribution and Precipitation Seasonality (bio_15) with a 33.70% contribution. Nevertheless, precipitation-based variables such as bio_12 and bio_15 align with the species’ ecology, reflecting its seasonal migratory behavior driven by these seasonal factors. The variables like Precipitation of the Coldest Quarter (bio_19) and Precipitation of the Driest Month (bio_14) likely influence their mating and nesting seasons, which occur during the dry season. Furthermore, the Euclidean Distance to Waterbodies (euc_river), contributing 8.53%, is another crucial habitat variable, as gharials inhabit waterbodies along with their riparian zones. These variables likely explain the identification of similar environmental envelopes which are anticipated to act as potential climate refugia in the future [82]. These findings underscore the dependence of gharials on climate shifts and their potential climatic resilience to withstand any climatic changes [83,84]. Additionally, if conservation initiatives focused on ensuring the survival of this species are directed towards these expanded future climatic zones, they are likely to contribute to an increase in its population viability [18]. Furthermore, the projected increase in habitat suitability for the studied species aligns with similar findings in other sympatric species, such as *Crocodylus porosus*, where the largest habitat expansions have been observed under high emission scenarios such as SSP585 [85]. It also provides further support for the notion that the species may not be directly impacted by climatic changes but could nonetheless be vulnerable to the indirect effects of anthropogenic interference, such as habitat alterations.

This reliance of the gharial and other reptiles on environmental conditions is well-documented in the previous research [86,87]. Such insights reinforce the critical need to integrate climate-based predictors into conservation strategies to ensure the long-term survival of any species. The observed increase in suitable habitat areas across various Indian states is accompanied by a significant rise in the number of habitat patches, as indicated by the metric of NP in the future climatic scenarios. The increase in NP, ranging from 44.32% to 96.13%, signifies the expansion and is critical for prioritizing conservation efforts for the gharial. The increase in the LPI by up to 33.94% suggests an enlargement of the individual habitat patches, indicating that these areas are not only more numerous but also larger in size. Moreover, the proximity between these habitat patches, as indicated by the AI, remains stable and suggests that the patches are spatially close to each other. This expansion of suitable habitat patches is vital for ensuring the persistence of the gharial population, as larger, more contiguous patches provide enhanced opportunities for breeding, foraging, and population connectivity [88,89]. Consequently, states with currently high suitability areas, such as Madhya Pradesh and Uttar Pradesh, which are expected to experience further growth, need to enhance and increase monitoring efforts for the gharial and its habitat. Over the years, gharial abundance has been rising in the upper Ganges and its tributaries (>19.6%), and the population in this central region is now stable [18,28]. Specifically, the Chambal River supports the highest number of breeding individuals (*n* ≈ 500), followed by the Girwa (*n* ≈ 50), Ramganga (*n* ≈ 32), and Gandak River (*n* ≈ 21) [29]. While habitat suitability in Uttarakhand has shown an increase, enhanced ground-level evaluation is required, as the rocky and rapid riverine areas may not be ideal for the species. However, changes in the river course could lead to ecologically viable habitats in the future. Additionally, states like Odisha and Rajasthan must prioritize and strengthen conservation and habitat protection for gharials, as they are predicted to lose habitat suitability in the coming years. This is crucial, given that gharials were once frequently sighted in the lower Ganges, but the population has since declined or shifted due to extensive anthropogenic pressures. Furthermore, the centroid shift towards eastern India supports the observation that the northeastern states have also seen an expansion in habitat suitability. Specifically, the Mahanadi and Brahmaputra rivers require additional surveys and ecological assessments to detect gharials and evaluate the viability of the region for their native population. Given the rapidly shifting climate conditions, it is essential to assess the connectivity between the identified suitable gharial habitats and populations. While the expansion of suitable areas is promising, inadequate connectivity between these patches could negatively impact the gharial population or other sympatric species. Thus, evaluating the connectivity between the identified suitable patches by extensive field surveys is recommended to ensure population viability and facilitate gene flow.

Furthermore, it is crucial to designate the suitable patches identified in this study as Protected Areas (PAs) similar to the National Chambal Sanctuary. Prior studies have shown that herpetofauna species currently distributed in PAs are likely to experience minimal decline or remain stable under future climate change [90]. Additionally, safeguarding the river and its surrounding areas within the identified suitable extent is essential for the breeding, foraging, and overall survival of gharials [38]. Hence, protecting these riverbeds and riparian areas, along with monitoring the growth of invasive plant species on them, is also vital for maintaining the habitat quality. Moreover, as the suitable habitat for the gharial expands in the future, there is a growing risk of possible conflict with humans. While gharials are not inherently dangerous to humans, they may be perceived similarly to more aggressive species, such as the mugger crocodile, potentially leading to fear and conflict [91]. The mugger crocodile poses a significant threat and competition to gharials, as it can directly compete for resources and, in some instances, attack gharial juveniles. Hence, to mitigate this threat, it is crucial to ensure vigilant monitoring and protection of gharial breeding areas. Moreover, during the breeding season, nesting female gharials guarding the nests may pose risk to humans and livestock, making the implementation of mitigation strategies essential to prevent human–gharial conflicts. Therefore, conducting awareness programs targeting local communities and other stakeholders near identified suitable habitats is critical for fostering coexistence and ensuring the gharial’s long-term survival [37]. This is particularly important because local fishing communities may view gharials as competitors for fish resources, and the designation of Protected Areas (PAs) could potentially impact their livelihoods. Such circumstances might lead to increased hostility, including hunting or killing of gharials which underscores the prioritized need of educating communities positively about this species especially in the identified extents.

Moreover, monitoring and curbing the illegal trade of gharial hatchlings involving both local populations and enforcement agencies is also imperative [92]. Additionally, the prevalence of harmful fishing practices, such as the use of nylon nets or “manjha”, poses a severe threat to both the gharial population and its prey base, which must be urgently controlled by local administration [93]. Furthermore, the sand mining or any anthropogenic activities in and around riverine systems near gharial habitats must also be regulated to protect critical breeding and basking sites. Thus, vigilant monitoring and targeted conservation efforts to mitigate such threats should be systematically directed towards the regions currently identified by the model, as well as the projected future ranges. This comprehensive approach would help ensure that potential climate refugia and present suitable habitats are adequately safeguarded. Notably, the erratic flow and degradation of water quality across gharial habitats are significant concerns, emphasizing the need for the maintenance of stable environmental conditions for the species’ survival. Hence, to enhance conservation efforts, in-depth ecological and genetic studies of the entire gharial population across its range are recommended, utilizing advanced technologies. The integration of artificial intelligence for individual identification, drone surveys, satellite tagging, VHF and acoustic surveys, etc., can provide crucial insights into gharial ecology and behavior. Specifically, it is recommended to conduct field surveys in the Mahanadi and Brahmaputra rivers for gharial detection, as well as to carry out genetic assessments of the native populations in these regions. Hence, to ensure the effective conservation of this species, it is imperative for the IUCN-SSC CSG, non-governmental organizations, leading bodies such as the Ministry of Environment, Forest and Climate Changes, Government of India, and other relevant peer institutions to establish coordinated and collaborative efforts with other state-level departments, fostering a unified approach to safeguarding extant populations. These necessary measures must be discussed across various public platforms to strengthen gharial conservation efforts in the landscape and prevent this critically endangered species from further decline or extinction.

## 5. Conclusions

The critically endangered *G. gangeticus*, native to the Indian subcontinent, is the sole surviving member of the Gavialidae family and serves as a keystone species in freshwater ecosystems. Despite its ecological significance, this species has suffered from a lack of comprehensive biological and ecological assessments over the past century, hindering the implementation of effective conservation initiatives to aid its recovery in the wild. Although some research has been conducted on gharials, there remains a significant knowledge gap regarding their responses to rapidly changing climatic conditions, particularly within their range in India. To address this research gap, the present study evaluates how gharials may adapt to such climatic shifts in this landscape. The study reveals that the extent of suitable habitat is projected to expand under future climate scenarios, corroborating patterns observed in other sympatric species. However, the identified suitable areas require prioritization and rigorous ground-truthing to determine their capacity to support sustainable gharial populations over the long term. The findings and recommendations outlined in this study provide an important and valuable framework for guiding future conservation efforts. Thus, by addressing habitat suitability and adaptation under shifting climatic conditions, this work contributes to securing the survival of gharials and promoting their continued existence within the dynamic landscapes of the Indian subcontinent.

## Figures and Tables

**Table 1:** Model fit metrics for each of the used modeling algorithms and for the final ensemble model for estimation of habitat suitability of gharial. The five model algorithms were used with the threshold of <0.75 AUC score. The models were Boosted Regression Tree (BRT), Maximum Entropy (MaxEnt), Random Forest (RF), Generalized Linear Model (GLM), and Multivariate Adaptive Regression Splines (MARS). AUC: Area under Curve, ΔAUC: Change in Area under curve (Training–Cross Validation), PCC: Proportion Correctly Classified, TSS: True Skill Statistic.

Model	Dataset	AUC	ΔAUC	PCC	TSS	Kappa	Specificity	Sensitivity
BRT	Train	0.993	0.04	94.7	0.893	0.869	0.946	0.948
	CV	0.953		90.5	0.798	0.771	0.886	0.912
GLM	Train	0.944	0.037	89.6	0.789	0.75	0.892	0.898
	CV	0.907		88.2	0.745	0.713	0.852	0.892
MARS	Train	0.973	0.017	90.5	0.81	0.771	0.905	0.905
	CV	0.956		89.8	0.775	0.75	0.865	0.91
MaxEnt	Train	0.959	0.022	91.4	0.823	0.791	0.905	0.917
	CV	0.937		89.6	0.786	0.749	0.886	0.9
RF	Train	0.98	0.001	94	0.879	0.852	0.939	0.94
	CV	0.979		95.1	0.874	0.875	0.907	0.968

**Table 2:** The mean percentage contribution of the variables generated from the final model for gharial.

Variables	Abbreviations	BRT	GLM	MARS	MAXENT	RF	μ (Mean)	μ (Mean) %
Annual Precipitation	bio_12	0.271	0.260	0.177	0.165	0.054	0.185	33.75
Precipitation of Driest Month	bio_14	0.000	0.120	0.000	0.073	0.000	0.039	7.04
Precipitation Seasonality	bio_15	0.185	0.287	0.206	0.224	0.023	0.185	33.70
Precipitation of Coldest Quarter	bio_19	0.000	0.105	0.057	0.050	0.007	0.044	8.00
Mean Diurnal Range	bio_2	0.000	0.141	0.015	0.016	0.000	0.035	6.29
Isothermality	bio_3	0.000	0.000	0.006	0.001	0.000	0.002	0.28
Euclidean Distance to Waterbodies	euc_river	0.000	0.113	0.049	0.068	0.005	0.047	8.53
Human Influence Index	hum_foot	0.000	0.051	0.004	0.011	0.000	0.013	2.40

**Table 3:** The table represents the suitable area for gharials (in sq. km) across range Indian states under the present and different future climatic scenarios. SSP: Shared Socioeconomic Pathways. The suitable area is calculated based on the pixels categorized as ‘5’, with unanimous agreement of all five-participating models.

State	Present	SSP245 (2041–2060)	SSP245 (2061–2080)	SSP585 (2041–2060)	SSP585 (2061–2080)
Uttar Pradesh	10,954	5713	14,655	5682	19,699
Madhya Pradesh	10,797	18,708	25,027	32,241	31,150
Rajasthan	7074	5883	6320	6246	4254
Uttarakhand	5785	10,913	24,116	12,121	9501
Assam	1489	2576	3946	2167	6974
Bihar	1062	3008	3064	1354	4390
Orissa	111	0	0	54	4
West Bengal	97	87	487	767	721
Haryana	70	1905	2418	2699	3659
Sikkim	30	264	469	460	720
Himachal Pradesh	12	22	272	339	15
Arunachal Pradesh	6	412	465	283	1283
Manipur	0	1252	2878	667	2213
Jharkhand	0	0	412	0	3239
Chhattisgarh	0	0	0	101	1809
Nagaland	0	328	314	330	1378
Mizoram	0	1	27	23	802
Meghalaya	0	45	87	92	93
Delhi	0	25	158	72	0

**Table 4:** The table represents the habitat quality and geometry of the suitable areas within the training area in present and future climatic scenarios. SSP: Shared Socioeconomic Pathways; NP: Number of patches; PD: Patch density; LPI: Largest patch index; TE: Total edge; LSI: Landscape shape index; AI: aggregate index.

Scenario	NP	PD	LPI	TE	LSI	AI
Present	388	3,956,579	1.0256	119.344	19.8265	90.3325
SSP245 (2041–2060)	560	5,716,036	1.102	202.408	26.8187	90.4336
SSP245 (2061–2080)	634	6,471,370	1.2852	266.928	29.1225	90.519
SSP585 (2041–2060)	555	5,665,000	1.6909	235.224	27.5614	90.3487
SSP585 (2061–2080)	761	7,809,127	1.3737	321.296	31.548	90.7826

## Data Availability

The original contributions presented in this study are included in the article/Supplementary Material. Further inquiries can be directed to the corresponding authors.

## Supplementary Materials

The following supporting information can be downloaded at: www.mdpi.com/article/10.3390/ani15060896/s1; Figure S1. Figure showing the correlation (<0.8) between the covariates chosen for final model for gharial. The Pearson correlation coefficient is the primarily used here. However, if the Spearman or Kendall correlation coefficient exceeds the Pearson correlation coefficient, an “s” or “k” will be displayed in the bottom-right corner of the variable box. Figure S2. Confusion matrixes, Residual plots and Model Calibration plots for gharial. Row 1 represents spatial pattern of residuals where color ramp indicates the magnitude of deviance and size represents the quantity. Row 2 represents the model calibration plot across all five selected models for cross-validation split. Row 3 represents a confusion matrix for the selected five models, plotted by observed vs. predicted where color ramp from lowest value 0% (white) to 100% (red) indicates the quantification of particular pair types. Column a. represents plots for BRT, Column b. represents plots for GLM, Column c. represents plots for MARS, Column d. represents plots for MaxEnt and Column e. represents plots for RF model. Figure S3. Evaluation Matrix performance across model runs for gharial. Brown—represents the correlation coefficient among the five different models. Yellow—represents the proportion of deviance explained; Green—represents the Proportion of correctly classified; Blue—represents Area under curve (AUC) and Pink—represents true skill statistics. Table S1. GPS locations of gharial presence during the primary survey for this study. Table S2. The table represents the total suitable area (in sq. km) for gharial within the training area under the present and future climatic scenarios.
