# Supplementary material for: Riverine Realities: Evaluating Climate Change Impacts on Habitat Dynamics of the Critically Endangered Gharial (Gavialis gangeticus) in the Indian Landscape
Source: Animals (Basel). 2025 Mar 20;15(6):896. doi: 10.3390/ani15060896 (PMC11939341; doi:10.3390/ani15060896)
Supplement: Supplementary file 1 [file animals-15-00896-s001.zip › animals-3497816-supplementary.pdf]

Supplementary Information

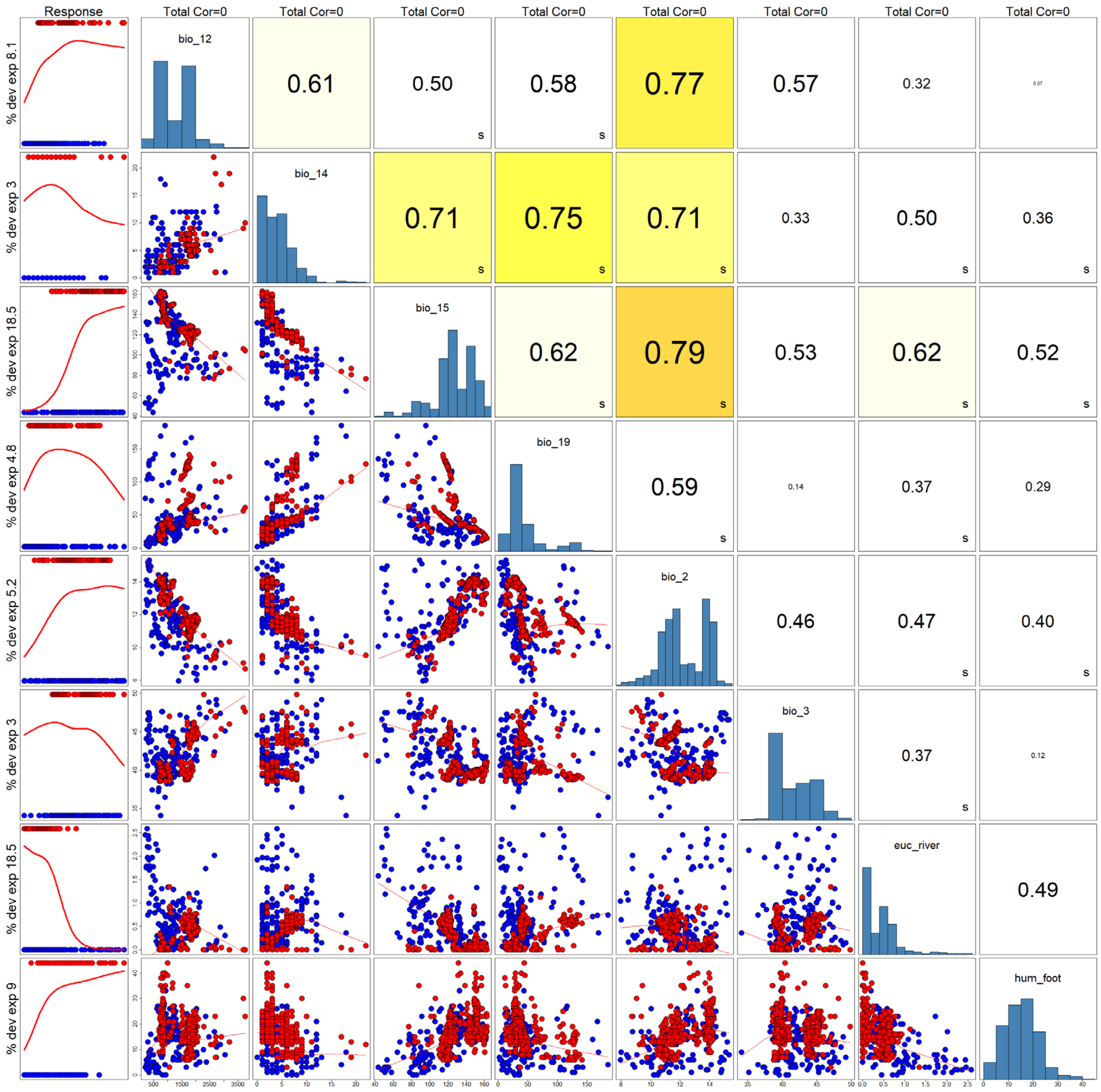

Figure S1. Figure showing the correlation (<0.8) between the covariates chosen for final model for gharial. The Pearson correlation coefficient is the primarily used here. However, if the Spearman or Kendall correlation coefficient exceeds the Pearson correlation coefficient, an "s" or "k" will be displayed in the bottom-right corner of the variable box.

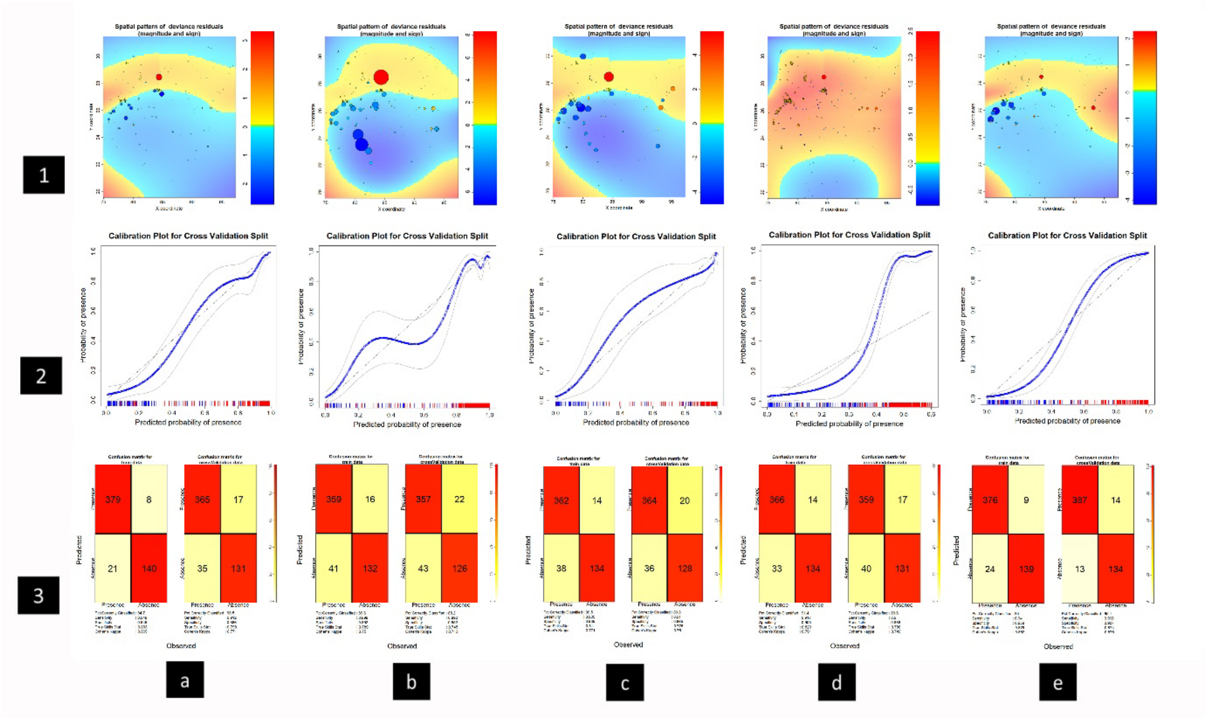

Figure S2. Confusion matrixes, Residual plots and Model Calibration plots for gharial. Row 1 represents spatial pattern of residuals where colour ramp indicates the magnitude of deviance and size represents the quantity. Row 2 represents the model calibration plot across all 5 selected model for cross-validation split. Row 3 represents confusion matrix for selected 5 models, plotted by observed vs. predicted where colour ramp from lowest value 0% (white) to 100% (red) indicates the quantification of particular pair types. Column a. represents plots for BRT, Column b. represents plots for GLM, Column c. represents plots for MARS, Column d. represents plots for MaxEnt and Column e. represents plots for RF model.

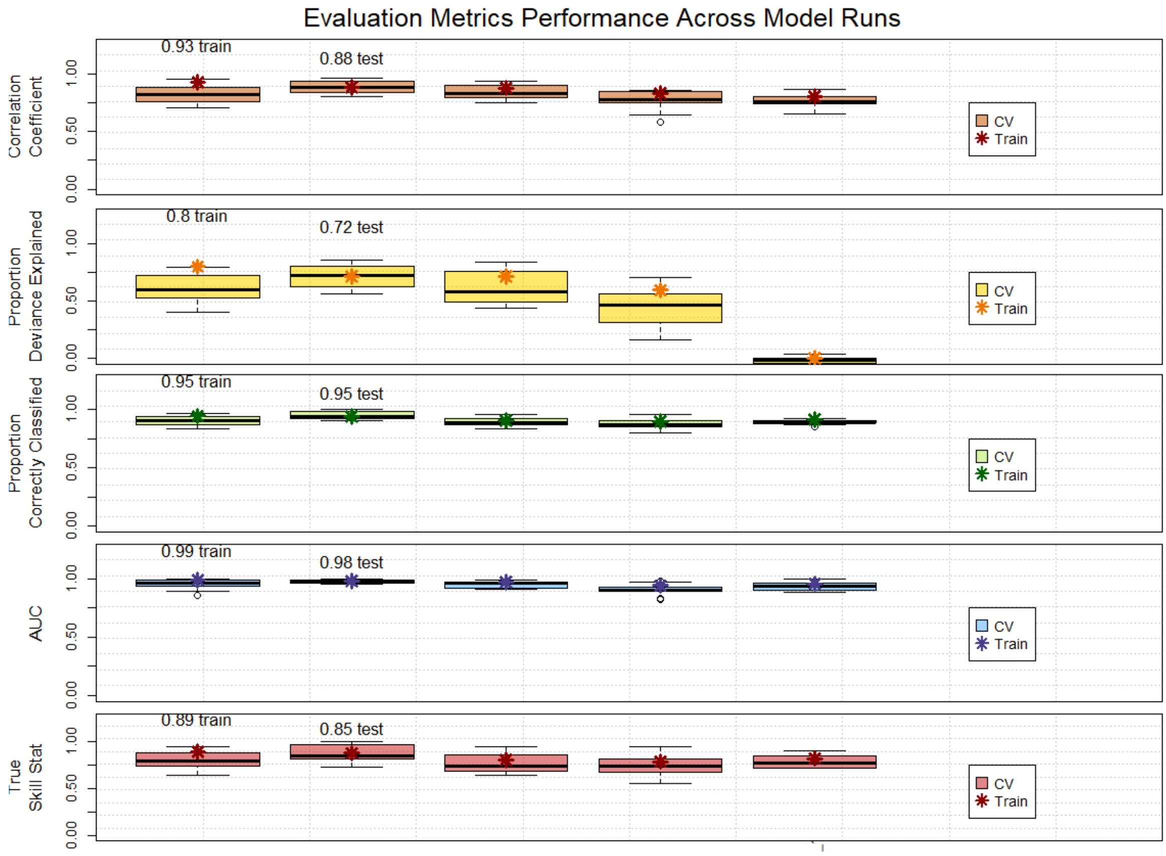

Figure S3. Evaluation Matrix performance across model runs for gharial. Brown - represents the correlation coefficient among the 5 different models. Yellow - represents the proportion of deviance explained; Green - represents the Proportion of correctly classified; Blue - represents Area under curve (AUC) and Pink - represents true skill statistics.

Table S1. GPS locations of gharial presence during the primary survey for this study.

Latitude	Longitude	River
29.370789	78.035004	Upper Ganges
27.523785	81.352174	Ghaghara River
27.506765	81.370964	
27.488638	81.377645	
27.461405	81.376854	
27.225746	81.464836	
26.886995	81.769012	
26.808325	81.944023	
26.789698	81.968529	
26.825785	82.164093	
25.98183	76.80333	Chambal River
26.22802	77.12527	
26.39472	77.43475	
26.50314	77.67454	
26.61263	77.88078	
26.65907	77.90371	
26.68178	77.98841	
26.74008	78.11059	
26.27694	77.27977	
26.53853	77.75400	
26.54594	77.76593	
26.71709	78.09190	
26.66499	77.96574	
26.76078	78.78394	
26.76452	78.80649	
26.76332	78.79797	
26.69956	78.86781	
26.70008	78.86683	
26.77881	78.73904	
26.77757	78.73797	
26.65848	78.94827	
26.65119	78.99717	
26.59157	78.03719	
26.52676	79.15494	
26.47872	79.23030	
26.49027	79.24606	

Table S2. The table represents the total suitable area (in sq. km.) for gharial within the training area under the present and future climatic scenarios.

Scenario	Area
Present	37487
SSP 245 (2041-2060)	51142
SSP 245 (2061-2080)	85115
SSP 585 (2041-2060)	65698
SSP 585 (2061-2080)	91904
